# Comprehensive urinary metabolomic profiling and identification of potential noninvasive marker for idiopathic Parkinson’s disease

**DOI:** 10.1038/srep13888

**Published:** 2015-09-14

**Authors:** Hemi Luan, Liang-Feng Liu, Zhi Tang, Manwen Zhang, Ka-Kit Chua, Ju-Xian Song, Vincent C.T. Mok, Min Li, Zongwei Cai

**Affiliations:** 1 Department of Chemistry, Hong Kong Baptist University, Hong Kong SAR, China; 2 School of Chinese Medicine, Hong Kong Baptist University, Hong Kong SAR, China; 3 Mr. & Mrs. Ko Chi-Ming Centre for Parkinson’s Disease Research, Hong Kong Baptist University, Hong Kong SAR, China; 4 Department of Medicine and Therapeutics, Faculty of Medicine, The Chinese University of Hong Kong, Hong Kong SAR, China

## Abstract

Urine metabolic phenotyping has been associated with the development of Parkinson’s disease (PD). However, few studies using a comprehensive metabolomics approach have investigated the correlation between changes in the urinary markers and the progression of clinical symptoms in PD. A comprehensive metabolomic study with robust quality control procedures was performed using gas chromatography - mass spectrometry (GC - MS) and liquid chromatography - mass spectrometry (LC - MS) to characterize the urinary metabolic phenotypes of idiopathic PD patients at three stages (early, middle and advanced) and normal control subjects, with the aim of discovering potential urinary metabolite markers for the diagnosis of idiopathic PD. Both GC-MS and LC-MS metabolic profiles of idiopathic PD patients differed significantly from those of normal control subjects. 18 differentially expressed metabolites were identified as constituting a unique metabolic marker associated with the progression of idiopathic PD. Related metabolic pathway variations were observed in branched chain amino acid metabolism, glycine derivation, steroid hormone biosynthesis, tryptophan metabolism, and phenylalanine metabolism. Comprehensive, successive metabolomic profiling revealed changes in the urinary markers associated with progression of idiopathic PD. This profiling relies on noninvasive sampling, and is complementary to existing clinical modalities.

Parkinson’s disease (PD) is a multisystem neurodegenerative disorder which afflicts nearly 1% of people above the age of 60^1^. The loss of dopaminergic neurons in the substantia nigra pars compacta (SNpc)^2^ gives rise to the characteristic motor disturbances that include bradykinesia, resting tremor and rigidity. For pathological confirmation, autopsy-confirmed pathologic Lewy body has been considered as the diagnostic standard for PD^3^, but there are currently no blood or laboratory tests to clearly identify PD in clinical practice. Signs and symptoms are often used for evaluation and diagnosis of PD. However, early signs and symptoms of PD may be mild and considered as the consequence of normal aging. Growing evidence suggests that decline in physical and mental health begin several years before confirmed diagnosis^4^,^5^,^6^. Many risk factors of PD such as aging^7^ and environmental toxins^8^ are likely to contribute to the pathogenesis of PD by initiating chronic changes throughout the body. Subsequent alterations in energy metabolism, oxidative stress, inflammation, and corticosteroid signaling occur that could further contribute to the development of PD^9^,^10^,^11^. Given the effective interventions for delaying or preventing the loss of dopaminergic neurons in PD patients^12^, early identification of individuals at risk is particularly crucial.

Metabolic profiling has been introduced into PD research and shows great potential value for the study of the pathophysiological changes associated with or resulting from the disease. Metabolomics is sensitive for detecting biochemical changes, including those caused by environmental and genetic factors, and therefore can characterize complex phenotypes and biomarkers of specific physiological responses^13^. Several studies have explored metabolic anomalies in PD. They have suggested that disturbances in the metabolic pathways related to oxidative stress, energy metabolism and neurotransmitters are associated with the progression of PD^14^,^15^,^16^,^17^. These observations raise the possibility that alterations in urine metabolite signatures could indicate the onset of PD in its earliest stage. Because urine contains most of the body’s metabolic end products, and because it entails noninvasive sampling, urine has been a “favored” marker source for disease research^18^. Comprehensive and unbiased coverage of urinary metabolites may allow us to characterize the dynamic metabolic phenotypes of PD. In our previous study, LC-MS-based urinary metabolite profiling revealed profound abnormality in the metabolic processes of PD patients, and the extent of the abnormality correlated with the severity of PD^19^. Michell *et al.* also reported changes in urine composition of PD patients, and suggested that these changes may be more helpful for predicting PD than changes in serum^15^. Here, we report a comprehensive metabolomic profiling using GC-MS and LC-MS technology, with the goal of identifying urinary metabolite markers that can be used for evaluate the development of PD.

## Results

### Clinical data and urine metabolic profiles

The clinical information of this study is given in Table 1. Of the 157 urine samples, 92 samples were collected from PD patients (aged 40–80 years) and 65 samples were collected from normal control subjects (aged 54–76 years). In the PD group, 14 (15.2%) patients had early-stage idiopathic PD; 59 (64.1%) patients had mid-stage idiopathic PD; and 19 (20.7%) patients had advanced-stage idiopathic PD according to the Hoehn and Yahr scale rating system. There were no significant variations of biochemical markers among the patients in different stages of PD (Table 1).

**Figure 1:**
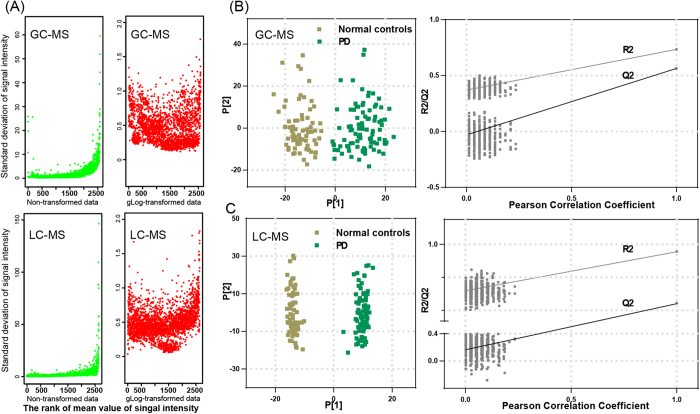
Variance stabilization and OPLS-DA analysis of metabolite profiles. (**A**) Standard deviations *vs* rank of mean intensities for urine samples. Each dot represents one peaks. Peaks are sorted by increasing mean intensities calculated on XCMS output. (**B**) Scores plot of the OPLS-DA model (left) and the corresponding permutation test (n = 500, right) for urine metabolic profile analyzed by using GC-MS. (**C**) Scores plot of the OPLS-DA model (left) and the corresponding permutation test (n = 500, right) for urine metabolic profile analyzed by using LC-MS.

We obtained 2581 (95.8%) and 2790 (74.5%) retention time-exact mass pairs in each sample profile by GC-MS and LC-MS, respectively. As showed in Fig. 1A, most of the higher peak intensities in metabolic profiles exhibited larger variability. To reduce the variation in peak intensity, which increased with the rank of mean intensity during MS analysis, the GC-MS and LC-MS profiles were processed by applying gLog-transformation, which successfully stabilized the variance across the intensity range.

PCA score plot representation of QC samples showed no drift during the GC-MS and LC-MS analysis (See Supplemental Figures S1 and S2). Thus, reproducibility and stability of metabolic features were acceptable and subsequently used for statistical analysis. The well-established OPLS-DA model demonstrated satisfactory modeling for GC-MS (R2X = 0.63, R2Ycum = 0.85, Q2cum = 0.60) and LC-MS (R2X = 0.43, R2Ycum = 0.99, Q2cum = 0.87). Both OPLS-DA score plots showed the normal controls are clearly separated from the PD group in the first component (P[1]). This separation clearly demonstrates the difference in urinary metabolite levels that exists between PD and normal control subjects (Fig. 1B,C, left). Two permutation tests (n = 500) were also performed to validate the two OPLS-DA models (Fig. 1B,C, right). The R2 and Q2 values of the original OPLS-DA models were higher than the randomly classified permutation distribution; this shows that the two original OPLS-DA models are valid.

### Differentially expressed metabolites for three stages of PD

Urinary metabolites passing the VIP threshold (VIP > 1) in the above-mentioned two OPLS-DA models and the Mann-Whitney U test (P < 0.05) after FDR correction were selected. Statistically, the differences are significant enough to discriminate PD patients from normal controls. 19 metabolites and 27 metabolites identified by GC-MS and LC-MS, respectively, were significantly altered in PD patients (Table 2). These metabolites, annotated by the Kyoto Encyclopedia of Genes and Genomes (KEGG) database, represent key metabolic pathways involving branched chain amino acid metabolism, glycine derivation, tryptophan metabolism, phenylalanine metabolism, lysine metabolism, histidine metabolism, citrate cycle and steroid hormone biosynthesis. Of the altered metabolites, 34 showing a significant difference in levels (P < 0.05) compared with normal control subjects were shared by all three types of PD patients. 10 metabolites, namely coumaric acid, tryptophan, tyrosine, succinic acid, pimelic acid, lysine, hypoxanthine, pyridoxic acid, glutaric acid and hexanoylglycine, were significantly altered in mid- and advanced- stages PD. Indoleacetic acid was significantly altered in early- and mid- stages PD. Aspartic acid was significantly disturbed only in mid-stages PD. Variations of these metabolites were expressed as -fold change (FC) in PD patients from early-stage to advanced-stage relative to normal controls (Table 2).

### Evaluation of metabolic markers for PD

**Figure 2:**
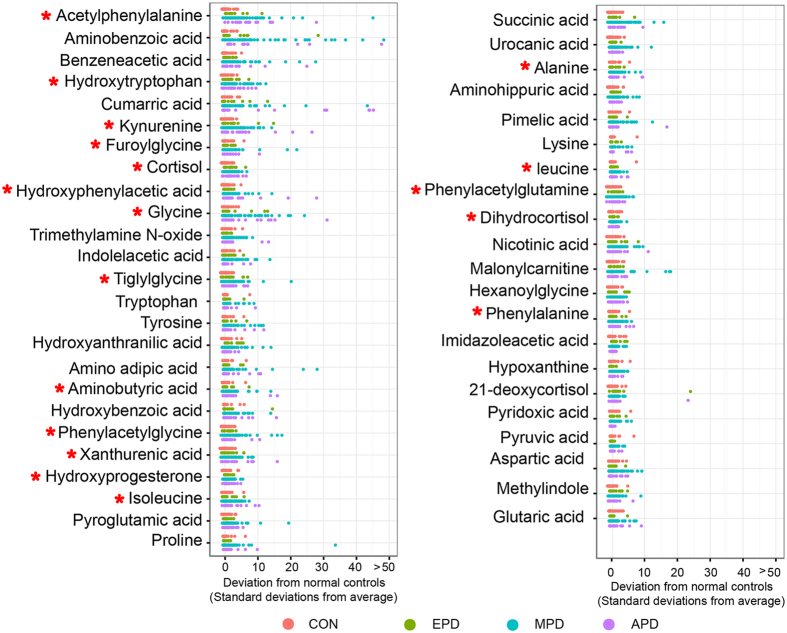
Z-score plot of 46 metabolites altered in PD patients relative to the mean in normal controls. Each point represents one metabolite in one sample, colored according to disease stage (red, normal controls (CON); green, early-stage PD (EPD); blue, mid-stage PD (MPD); purple, advanced-stage PD (APD)). The horizontal axis has been truncated at 50 standard deviations. Red asterisks (*) denote the statistical significances between the early-stage PD subjects and controls.

As shown in the Table 2, the combination of multivariate and univariate analysis was performed, and it identified 46 differential metabolites for discriminating PD patients from control subjects. The relative distribution of these 46 differential metabolites across PD groups and normal controls is presented in the z-score plots (Fig. 2). These 46 differential metabolites monitored in patients’ samples were normalized to the means of the normal control samples. The plots showed metabolic alterations in PD patients (z-score range: −1.53 to 183.65) compared to normal control subjects (z-score range: −1.53 to 7.75).

**Figure 3:**
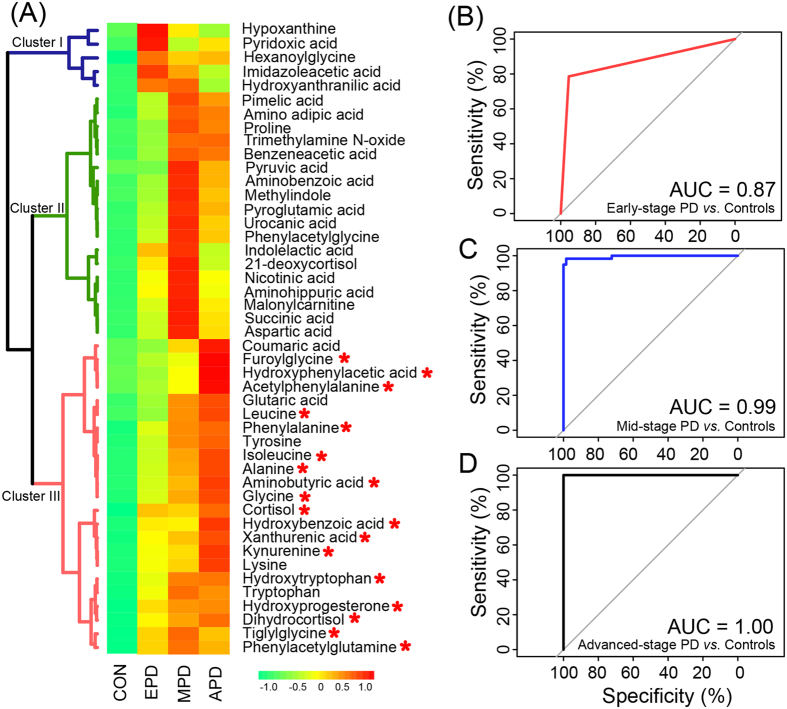
Evaluation of metabolic marker for PD. (**A**) Heatmap shows mean intensity of differential metabolites in normal controls (CON), early-stage PD (EPD), mid-stage PD (MPD) and advanced-stage PD (APD). Shades of green to red represent increasing mean value of a metabolite. Red asterisks (*) denote the statistical significances between the early-stage PD and controls. (**B**–**D**) ROC curves of a logistic regression model for distinguishing early-stage PD (**B**), mid-stage PD (**C**), and advanced-stage PD (**D**) from normal controls using the above mentioned 18 metabolites.

In order to clearly visualize the stage-dependent variations, mean intensities of differential metabolites in the control group, early-stage PD group, mid-stage PD group and advanced-stage PD groups were used to generate a heat map (Fig. 3A). Three major clusters were constructed based on the differential metabolites. The cluster I consisted of five metabolites that had the increased level in early stage PD. 18 metabolites included in the cluster II had the increased level in middle stage PD. The heat map indicates that the progressive increase of mean intensity in the cluster III at the bottom (red color) should be associated with disease stages of PD. 18 metabolites in cluster III of the heat map had statistical significance in the early-stage PD group compared to controls (Fig. 3). Z-score plot (Fig. 2) showed fewer alterations in 18 metabolites in early-stage PD patients (z-score range: −0.16 to 14.43) compared to mid- and advanced- stage PD patients (z-score range: −0.38 to 45.07, mid-stage; −0.05 to 87.38), but higher metabolic alterations compared to normal control subjects (z-score range: −1.53 to 7.75). These 18 metabolites were: acetylphenylalanine, hydroxytryptophan, kynurenine, furoylglycine, cortisol, hydroxyphenylacetic acid, glycine, tiglylglycine, aminobutyric acid, hydroxybenzoic acid, xanthurenic acid, hydroxyprogesterone, isoleucine, alanine, leucine, phenylacetylglutamine, dihydrocortisol and phenylalanine. ROC curves of a logistic regression model were constructed by using the above-mentioned 18 metabolites. The area under-ROC curves (AUC) values of 0.87 indicated high predictive ability for early-stage PD patients and control subjects (Fig. 3B). The higher AUC values of 0.99 and 1.00 were obtained from the curves created from the data from mid- and advanced stage PD, respectively (Fig. 3C,D).

## Discussion

This study employed GC-MS and LC-MS for comprehensive metabolomic profiling of metabolites in urine of 92 idiopathic PD patients and 65 normal control subjects. OPLS-DA models based on metabolic profiles were constructed and able to discriminate all of the PD patients from the control subjects; Levels of 46 metabolites were found disturbed in PD patients (Fig. 1 and Table 2). 22 differential metabolites were reported both in our previous LC-MS-based study and present study^19^, and 24 differential metabolites was newly identified in present study (**See** Supplemental Table S1). In this study, we were able to enlarge the metabolite profiles detected from GC-MS and LC-MS based platforms and further evaluate the discrimination ability of urinary metabolites in the different disease stages of PD. We identified 18 metabolites out of the above-mentioned 46 differential metabolites that showed progressive increases of mean concentration correlating with the different disease stages of PD. The combination of 18 metabolites not only had high discrimination ability for the early-stage PD (AUC = 0.87, Fig. 3B), but also accurately distinguished the mid- and advanced- stages PD patients from control subjects (AUC = 0.99, Fig. 3C; AUC = 1.00, Fig. 3D).

**Figure 4:**
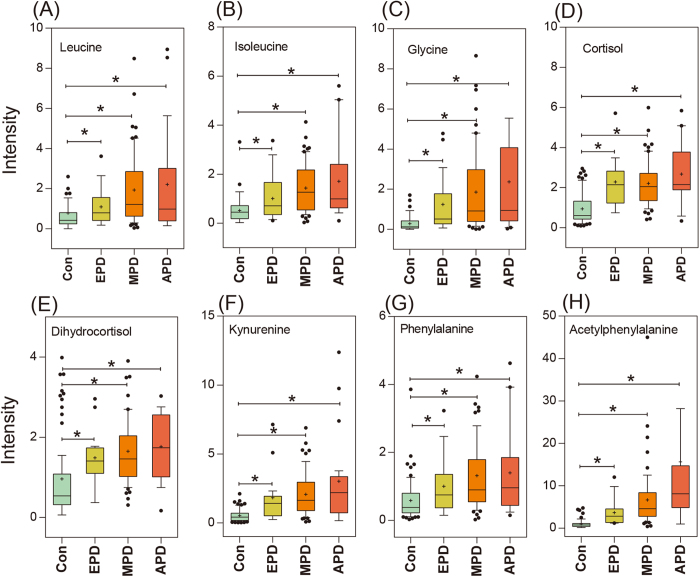
Box plot showing levels of representative metabolites in normal controls (CON), early-stage PD (EPD), mid-stage PD (MPD) and advanced-stage PD (APD). (**A**) leucine, (**B**) isoleucine, (**C**) glycine, (**D**) cortisol, (**E**) dihydrocortisol, (**F**) kynurenine, (**G**) phenylalanine; (**H**) acetylphenylalanine. Asterisk(*) denotes P < 0.05. A cross (+) denotes the mean value of the data.

These findings indicate that 18 metabolites show great promise as metabolite markers for evaluating PD, with related metabolic pathway variations observed in branched chain amino acid metabolism, glycine derivation, steroid hormone biosynthesis, tryptophan metabolism, phenylalanine metabolism. As showed in Table 2, increased excretion of branched-chain amino acids (leucine and isoleucine) was observed in the urine of idiopathic PD patients compared with that of controls (Fig. 4 and Table 2). The levels of leucine and isoleucine in the urine were positively correlated with the stage of PD. Branched-chain amino acids (BCAAs) play important roles in protein synthesis, energy production and synthesis of neurotransmitter glutamate in skeletal muscles, adipose tissue and brain^20^,^21^. Several early studies have showed that PD patients have slightly decreased concentrations of leucine and isoleucine in their CSF and plasma. Deficiency of leucine and isoleucine may contribute to muscle wasting, twitching and tremors^22^,^23^.

A group of glycine and glycine derivatives was significantly altered in the urine of PD patients, including glycine, furoylglycine, tiglylglycine and hexanoylglycine. It was reported that glycine could stimulate the release of dopamine and acetylcholine from tissue^24^,^25^. An increased level of glycine was also observed in the plasma and CSF from PD patients, which was consistent with the changes of glycine levels in urine of PD patients (Fig. 4 and Table 2)^22^. Urinary furoylglycine and tiglylglycine were significantly increased in patients with early-stage PD (Table 2). Furoylglycine, tiglylglycine and hexanoylglycine are products of the catabolism of fatty acids, which are associated with mitochondrial fatty acid beta-oxidation^26^.

Urinary excretion of cortisol is regarded as an indicator of increased oxidative stress, which contributes to dopamine cell degeneration in PD^27^. The significantly increased levels of serum cortisol were found in patients with advanced PD. Our data shows elevated levels of urinary cortisol, dihydrocortisol, hydroxyprogesterone and 21-deoxycortisol, indicating altered steroid hormone biosynthesis (Fig. 4 and Table 2). The increased levels of urinary cortisol, dihydrocortisol and hydroxyprogesterone were observed in the all stages of PD while urinary 21-deoxycortisol was only significantly altered in the mid- and advanced- stages PD (P < 0.05, Wilcoxon − Mann U test, Table 2).

Differentially expressed metabolites involved in tryptophan metabolism and phenylalanine metabolism were observed in the current study and our previous study. The level of urinary tryptophan catabolites involving kynurenine, hydroxytryptophan and xanthurenic acid were significantly elevated in patients with early-stage PD (Fig. 4 and Table 2). Changes in levels of tryptophan catabolites were related to mitochondrial disturbances and impairment of brain energy metabolism involved in the development of neurodegenerative disease^28^. Furthermore, an increased ratio of kynurenine to tryptophan was observed in PD patients. The enhanced degradation of tryptophan may be associated with the activated cell-mediated immune response typical of PD^29^. Altered phenylalanine, hydroxyphenylacetic acid, acetylphenylalanine, and phenylacetyglutamine levels indicate disturbed phenylalanine metabolism in early-stage PD (Fig. 4 and Table 2). Phenylalanine not only participates in protein sequence in all tissues, but is also a precursor for dopamine^30^. In the previous study, the levels of plasma phenylalanine were slightly increased without statistical significance^22^. Molina *et al.*^31^ reported cerebrospinal fluid tyrosine and phenylalanine levels in PD patients treated with levodopa were higher than those not treated with levodopa and also than controls, whereas other amino acids levels were unchanged. There were also previous studies showed tyrosine and phenylalanine levels in cerebrospinal fluid of patients with PD were unchanged^32^. More general and comprehensive studies of how PD drugs modify urinary amino acids are still need to be investigated. The increased excretion of hydroxyphenylacetic acid in the urine of PD patients was consistent with the former reports of Sandler *et al.*^33^, and may be associated with neurological disorders in general^34^.

Although the existence of distinct population in PD patients with differences in signs and symptoms that are related to different metabolic signatures could be constructed, one limitation of this study is the population size of the early-stage PD samples. However, the sufficient statistical power in this study was achieved, because all stages of PD samples were used for statistical significance analysis. The small size of the early-stage PD sample is due to the fact that early warning signs and symptoms of PD patients may be ignored as part of normal aging in the clinical practice. Furthermore, the comprehensive evaluation of some factors’ effects on potential markers, such as secondary PD, drug treatment, gender, BMI, diet, and other CNS disorders still needs to be further investigated.

## Conclusion

In summary, this study combined GC-MS and LC-MS technology to profile urinary metabolites in patients with early-, mid- and advanced-stage PD. From a panel of 46 differential metabolites compared between PD patients and control subjects, 18 metabolites emerged as a metabolic marker with diagnostic potential. Furthermore, investigation is warranted to explore whether genes and enzymes related to these metabolites could help to elucidate the biological mechanisms of how PD develops at the systems level.

## Materials and Methods

### Clinical samples

A total of 157 subjects, namely 92 idiopathic PD patients and 65 normal controls, were recruited at the Hong Kong Baptist University Chinese Medicine Specialty Centre. The study was approved by the Ethics Committee of the Hong Kong Baptist University’s Institutional Review Board. The methods were carried out in accordance with the approved guidelines. Written information was provided and informed consent was obtained from all subjects. Patients were clinically diagnosed with idiopathic Parkinson’s disease according to the United Kingdom Parkinson’ s Disease Brain Bank (UKPDBB) criteria^35^. The inclusion criteria were UKPDBB clinical diagnostic criteria, stable treatment with levodopa, Hoehn and Yahr scale rating from 1 to 4, and normal liver and renal function. Subjects in any one or more of the following categories were excluded from our analysis: atypical or secondary Parkinsonism, use of antidepressants, Mini-Mental State Examination (MMSE) <24, history of psychosis, or severe suicidal tendency. Volunteers without neurological or psychiatric problems were recruited as normal controls. The clinical diagnosis and blood examination reports of all patients are provided in Table 1. Samples were collected from all subjects using the same protocol as follows: After overnight fasting, morning midstream urine was collected in a polypropylene container, then aliquoted into an Eppendorf tube and stored at −80 °C for GC-MS and LC-MS analysis.

### Biochemistry tests

Blood biochemical assay was performed with an automatic biochemistry analyzer (Hitachi Ltd., Tokyo, Japan). Routine blood, liver and renal function markers were assessed.

### Urine sample preparation and analysis by GC-MS

Urine samples were preprocessed, extracted, and derivatized as previously reported^36^,^37^. Briefly, each urine sample was thawed at room temperature and centrifuged 5 min at 3000 g speed in an Eppendorf centrifuge. Twenty microliter of water containing 4-chlorophenylalanine (0.5 mg/mL, internal standard) was added into 100 μl of each sample. The solution was mixed with 100 μl of sodium hydroxide (1 mol/L), 160 μl of methanol and 40 μl of pyridine in a 10 ml glass centrifuge tube. The derivative reaction was started by adding 50 μl of methyl chloroformate (MCF) and the pooled mixture was then shaken for 30 s using a vortex. The derivative procedure was repeated with the addition of another 50 μl MCF. After the two successive derivatization steps, 300 μl of dichloromethane was added and shaken for 10 s for separating the MCF derivatives. After adjusting the pH value with 200 μl of sodium bicarbonate (50 mmol/L), the dichloromethane layer containing derivatives was isolated and dried with anhydrous sodium sulfate and subsequently subjected to GC/MS analysis.

GC-MS analysis was performed with an Agilent 6890N gas chromatograph coupled with a 5975B mass spectrometric detector. The column used for all analysis was a DB-5MS capillary column coated with 5% diphenyl cross-linked 95% dimethylpolysiloxane (30 m × 250 um i.d., 0.25 um film thickness; Agilent J&W Scientific, Folsom, CA). Solvent delay was set for 5 min. The measurements were made with electron impact ionization (70 eV) in the full scan mode (m/z 50–650). The oven temperature was initially held at 50 °C for 2 min. Thereafter the temperature was raised with a gradient of 6 °C/min until 180 °C was reached. Afterward, the temperature was raised with a gradient of 6 °C/min up to 260 °C and then increased to 300 °C at a rate of 20 °C/min. This temperature was held for 2 min. The injection temperature and the interface temperature were both set to 280 °C. The flow through the column was held constant at 1 ml He/min. The temperature of quadrupole and the ion source temperature were adjusted to 150 °C and 230 °C, respectively. The peak abundances of MCF derivatives were used to quantify the concentrations of the amino and nonamino organic acids in the samples. The majority of the metabolites detected were identified by commercially available compound libraries: National Institute of Standards and Technology (NIST) and reference compounds available.

### Urine sample preparation and analysis by LC-MS

Urine sample preparation for LC-MS analysis was performed as we previously reported^19^. Briefly, the urine samples were thawed at room temperature. 100 μl of each thawed urine sample was precipitated by 100 μl of methanol. The mixture was then centrifuged under 14000 g for 10 minutes at 4 °C, and the supernatant was used for LC-MS analysis.

Each 10 μL aliquot of extract was injected into a Shimadzu Prominence LC system (Shimadzu) coupled online to an LTQ Orbitrap Velos instrument (Thermo Fisher Scientific, MA, USA) set at 30000 resolution (at m/z 400). Both positive and negative ion modes were used for sample analysis. The mass scanning range was 50–1000 m/z and the capillary temperature was 350 °C. Nitrogen sheath gas was set at a flow rate of 30 L/min. Nitrogen auxiliary gas was set at a flow rate of 10 L/min. Spray voltage was set to 4.5 kV and 3.0 kV for positive or negative ion mode, respectively. The LC-MS system was run in binary gradient mode. Solvent A was 0.1% (v/v) formic acid/water and solvent B was 0.1% (v/v) formic acid/methanol. The flow rate was 0.2 ml/min. A C-18 column (150 × 2.1 mm, 3.5 μm, Agilent, USA) was used for all analysis. The linear gradient was as follows: 5% B at 0 min, 5% B at 5 min, 100% B at 8 min, 100% B at 9 min, 5% B at 18 min and 5% B at 20 min.

### Quality control approach for metabolomic profiling

To obtain high quality data comparable to the metabolomic profiling (GC-MS and LC-MS), a quality assessment strategy based on the periodic analysis of quality control (QC) samples together with the real samples was employed in this study^38^. The QC samples consisted of mixing equal volumes of urine obtained from 20 PD patients and 20 control subjects before sample preparation as they were aliquoted for analysis. This pooled QC sample was prepared as described for real samples and used to estimate a “mean” profile representing all the peaks detected during the MS analysis. At the beginning of run, five QC samples were advisable to equilibrate the analytical platform and then injected at regular intervals (e.g., every ten real samples) throughout the analytical run in order to provide data^39^. The repeatability of data can be assessed and the intra-variation also can be corrected using QC spectra as described below.

### Data analysis

MS data was analyzed following a previously published method^19^,^40^. GC-MS data was initially preprocessed using MetAlign software for noise filtering and baseline correction. The output files were further processed by using XCMS software implemented with the freely available R statistical language (v 2.13.1). For LC-MS data preprocessing, data pre-treatment including peak picking, peak grouping, retention time correction, second peak grouping and annotation of isotopes and adducts was performed using XCMS and CAMERA software. The XCMS output was a list of the ion intensities of each peak; this output was generated using retention time (RT) and the m/z data pairs as identifiers for each ion. To obtain consistent variables, the resulting matrix was further reduced by the 80% rule, i.e., by removing peaks with more than 80% missing values (those with ion intensity = 0). The analytical variation was corrected with the quality control-based robust LOESS signal correction (QC-RLSC) algorithm^39^. A threshold of 30% was set for the relative standard deviation (RSD) values of metabolites in the QC samples. This threshold was used for the assessment of repeatability in metabolomics data sets^19^. Generalized logarithm- (gLog-) transformation was performed to stabilize the variance in datasets before multivariate statistical analysis^41^. Principle component analysis (PCA) was performed on UV-scaled data to visualize general clustering of QC samples together with all samples on the scores plot.

The nonparametric univariate method, Mann–Whitney–Wilcoxon test, was applied to measure the significance of each peak in the different groups, with results adjusted for multiple testing using false discovery rates (FDR) correction. On the basis of a variable importance in the projection (VIP) from the cross-validated orthogonal partial least squares discriminant analysis (OPLS-DA) model, peaks responsible for the difference in the metabolic profile scan of groups can be selected^40^. The peaks identified by two latent variables of OPLS-DA model were validated at a univariate level using the FDR test from the R statistical toolbox with the critical p-value set to not higher than 0.05. The heat map was performed using the “pheatmap” package for R. These clusters can be generated by using Pearson correlation as distance measure and complete linkage as clustering method. Z-score plots and heat maps were used for visualizing class-specific patterns of differential metabolites. Coupling the receiver operating characteristic curve (ROC) with its area under the curve (AUC), a widely used method to estimate the diagnostic potential of a classifier in clinical applications, was performed using the “pROC” package for R^42^.

Compound annotation for LC-MS data was performed by comparing the MS/MS spectra and retention times of commercially available standard compounds or the accurate masses of compounds obtained from the Human Metabolome Database (www.hmdb.ca). Compound identification from GC-MS data was performed by comparing the mass spectral data with NIST database with a similarity of more than 70%. The commercially available standards were used to verify these metabolites.

## Additional Information

**How to cite this article**: Luan, H. *et al.* Comprehensive urinary metabolomic profiling and identification of potential noninvasive marker for idiopathic Parkinson's disease. *Sci. Rep.* **5**, 13888; doi: 10.1038/srep13888 (2015).

## Supplementary Material

Supplementary Information

## Figures and Tables

**Table 1:** Clinical information and characteristics of study subjects.

**Characteristics**	Normalcontrols (n=65)	**Idiopathic PD patients (n** = **92)**		
		**Early-stage**	**Mid-stage**	**Advanced-stage**
Male/female	27:38	6:8	41:18	8:11
Age	59.8 ± 3.7	62.9 ± 7.6	62.7 ± 7.8	62.3 ± 9.2
Hoehn and Yahr score	—	1.0 ~ 1.5	2.0 ~ 2.5	3.0 ~ 4.0
Total bilirubin (μmol/L)	—	9.9 ± 3.6	9.9 ± 3.7	9.9 ± 3.7
Alkaline phosphatase (U/L)	—	59.9 ± 14.6	59.6 ± 14.5	59.6 ± 15.0
AST/SGOT (U/L)	—	18.3 ± 5.0	18.7 ± 5.7	18.5 ± 5.2
ALT/SGPT (U/L)	—	16.2 ± 10.8	16.1 ± 10.5	15.9 ± 10.2
Gamma GT (U/L)	—	20.9 ± 10.7	20.7 ± 10.6	20.7 ± 10.2
Total protein (g/L)	—	73.4 ± 3.9	73.3 ± 3.9	73.4 ± 4.0
Albumin (g/L)	—	43.2 ± 2.5	43.2 ± 2.5	43.4 ± 2.4
Urea (mmol/L)	—	5.8 ± 1.4	5.8 ± 1.4	5.8 ± 1.4
Creatinine (μmol/L)	—	70.7 ± 13.3	70.9 ± 13.3	70.4 ± 13.3
Sodium (mmol/L)	—	139.3 ± 2.3	139.3 ± 2.3	139.3 ± 2.1
Potassium (mmol/L)	—	4.0 ± 0.3	4.00 ± 0.3	4.0 ± 0.3
Chloride (mmol/L)	—	104.8 ± 2.5	104.7 ± 2.5	104.7 ± 2.4
HCO3 (mmol/L)	—	25.4 ± 2.4	25.5 ± 2.4	25.5 ± 2.4

Means ± standard deviation were given for each variable and each group; The classification of patients with different stages of PD was according to Hoehn and Yahr scale rating system.

**Table 2:** List of altered metabolites between three stages of PD patients and normal control subjects.

**Metabolites**	**VIP**^a^	**Early-stage PD vs. Controls**			**Mid-stage PD vs. Controls**			Advanced-stage PD vs.Controls		
		**P value**^b^	**FC**^c^	**AUC**^d^	**P value**^b^	**FC**^c^	**AUC**^d^	**P value**^b^	**FC**^c^	**AUC**^d^
Acetylphenylalanine*^LC^	3.00	3.06E-06	3.59	0.90	1.13E-16	6.51	0.93	4.57E-10	15.29	0.97
Aminobenzoic acid*^GC^	2.34	2.22E-04	6.63	0.82	9.67E-16	25.43	0.92	1.33E-09	16.74	0.96
Benzeneacetic acid*^GC^	2.01	5.94E-04	2.22	0.79	2.39E-09	5.50	0.81	1.75E-04	5.18	0.78
Hydroxytryptophan*^LC^	1.96	3.17E-04	2.35	0.81	6.22E-12	3.29	0.86	1.01E-05	3.33	0.83
Coumaric acid^GC^	1.94	2.17E-01	3.50	0.61	2.16E-11	10.59	0.85	9.35E-04	19.32	0.75
Kynurenine*^LC^	1.92	5.66E-04	3.44	0.80	4.08E-12	3.91	0.86	3.67E-06	5.71	0.85
Furoylglycine*^LC^	1.92	2.23E-04	2.77	0.82	3.42E-08	3.91	0.79	3.56E-04	9.26	0.77
Cortisol*^LC^	1.86	7.47E-05	2.43	0.84	7.55E-11	2.35	0.84	1.76E-06	2.85	0.86
Hydroxyphenylacetic acid*^LC^	1.85	4.28E-03	1.66	0.75	3.34E-09	2.78	0.81	2.68E-06	5.85	0.86
Glycine*^GC^	1.76	1.62E-03	4.48	0.77	5.23E-11	6.70	0.84	1.76E-06	8.54	0.86
Trimethylamine N-oxide*^LC^	1.73	1.25E-02	1.54	0.71	1.70E-11	3.09	0.85	1.04E-04	3.13	0.79
Indolelacetic acid*^LC^	1.66	3.64E-03	2.64	0.75	4.29E-08	3.49	0.79	5.56E-02	1.88	0.65
Tiglylglycine*^LC^	1.63	3.08E-03	2.13	0.75	1.78E-10	2.54	0.83	7.29E-05	2.20	0.80
Tryptophan*^GC^	1.62	1.63E-01	2.09	0.38	1.89E-06	3.18	0.75	4.42E-02	2.77	0.65
Tyrosine*^GC^	1.56	1.38E-01	2.18	0.63	9.09E-08	3.64	0.78	1.86E-03	3.76	0.74
Hydroxyanthranilic acid*^LC^	1.54	3.92E-06	2.60	0.90	6.25E-10	2.69	0.82	1.13E-02	1.54	0.69
Aminoadipic acid*^GC^	1.51	1.04E-02	2.35	0.72	2.46E-09	4.63	0.81	7.01E-04	4.14	0.76
Aminobutyric acid*^GC^	1.47	7.15E-03	2.08	0.73	1.07E-08	2.79	0.80	4.99E-06	3.57	0.85
Hydroxybenzoic acid*^LC^	1.47	3.08E-03	4.30	0.75	3.08E-05	4.26	0.72	3.41E-04	6.68	0.77
Phenylacetylglycine*^LC^	1.45	6.62E-03	1.74	0.73	3.05E-09	3.00	0.81	7.87E-04	2.26	0.75
Xanthurenic acid*^LC^	1.44	3.49E-03	2.01	0.75	5.37E-08	2.25	0.78	5.09E-03	2.75	0.71
Hydroxyprogesterone^LC^	1.43	1.08E-04	1.83	0.83	1.10E-09	2.04	0.82	8.34E-05	2.07	0.80
Isoleucine*^GC^	1.4	3.25E-02	1.94	0.68	4.95E-09	2.77	0.80	5.07E-05	3.29	0.81
Pyroglutamic acid*^LC^	1.4	5.89E-03	1.45	0.74	8.57E-10	2.41	0.82	4.72E-04	1.96	0.77
Proline*^GC^	1.37	3.93E-02	1.54	0.68	8.16E-08	3.61	0.78	9.52E-05	3.21	0.80
Succinic acid*^GC^	1.36	6.00E-02	1.71	0.66	1.32E-08	3.04	0.80	8.01E-03	2.10	0.70
Urocanic acid*^LC^	1.36	8.33E-03	1.45	0.73	1.93E-09	2.37	0.81	1.14E-04	1.82	0.79
Alanine*^GC^	1.34	1.39E-02	1.94	0.71	6.35E-08	2.68	0.78	2.25E-04	3.22	0.78
Aminohippuric acid*^LC^	1.31	9.46E-04	1.60	0.78	7.80E-10	2.22	0.82	4.61E-03	1.56	0.71
Pimelic acid*^GC^	1.29	7.95E-02	1.62	0.65	2.21E-08	3.05	0.79	7.72E-03	2.54	0.70
Lysine*^GC^	1.27	2.66E-01	2.19	0.60	4.71E-05	2.62	0.71	4.30E-02	3.42	0.65
Leucine*^GC^	1.27	4.19E-02	1.39	0.67	3.34E-07	2.46	0.77	8.50E-04	2.82	0.75
Phenylacetylglutamine*^LC^	1.25	4.46E-04	1.79	0.80	2.30E-07	2.06	0.77	1.14E-04	1.88	0.79
Dihydrocortisol*^LC^	1.24	2.72E-03	1.55	0.76	5.84E-08	1.72	0.78	3.14E-04	1.84	0.77
Nicotinic acid*^LC^	1.22	1.45E-02	1.63	0.71	3.86E-11	2.34	0.84	5.09E-03	1.65	0.71
Malonylcarnitine*^LC^	1.18	1.78E-02	1.51	0.70	1.34E-05	2.35	0.73	1.67E-03	1.70	0.74
Phenylalanine*^GC^	1.16	3.36E-02	1.71	0.68	4.82E-07	2.25	0.76	6.48E-04	2.40	0.76
Imidazoleacetic acid*^LC^	1.15	4.33E-05	1.86	0.85	3.09E-10	1.64	0.83	5.32E-04	1.29	0.76
Hypoxanthine*^LC^	1.13	6.73E-02	5.44	0.66	2.56E-06	2.96	0.75	2.76E-03	1.70	0.73
21-deoxycortisol*^LC^	1.1	1.34E-04	1.84	0.83	3.62E-08	2.60	0.79	2.25E-04	1.54	0.78
Pyridoxic acid*^LC^	1.1	1.77E-03	4.30	0.77	9.43E-07	1.92	0.76	2.54E-02	2.68	0.67
Pyruvic acid*^GC^	1.09	1.35E-01	1.02	0.63	7.18E-06	1.95	0.73	2.09E-02	1.61	0.68
Aspartic acid*^GC^	1.08	9.38E-02	1.50	0.64	1.73E-06	2.59	0.75	6.75E-02	1.82	0.64
Methylindole^LC^	1.06	1.27E-04	1.74	0.83	9.55E-15	1.76	0.90	1.53E-09	1.61	0.96
Glutaric acid*^GC^	1.03	5.66E-02	1.36	0.66	4.01E-07	2.09	0.76	7.87E-04	2.33	0.75
Hexanoylglycine*^LC^	1.02	8.65E-02	1.78	0.65	4.27E-06	1.60	0.74	7.28E-03	1.64	0.70

Asteriks (*) denotes metabolites are verified by reference standards. Superscript letter GC or LC indicated metabolites were detected with GC-MS or LC-MS platforms, respectively.

^a^Variable importance in the projection (VIP) was obtained from OPLS-DA model with a threshold of 1.0.

^b^P-value were calculated from Wilcoxon − Mann U test.

^c^Fold change (FC) was obtained by comparing those metabolites in PD group to control group.

^d^AUC: area under the ROC curve.
