# Supplementary material for: Comprehensive urinary metabolomic profiling and identification of potential noninvasive marker for idiopathic Parkinson’s disease
Source: Sci Rep. 2015 Sep 14;5:13888. doi: 10.1038/srep13888 (PMC4568456; doi:10.1038/srep13888)
Supplement: Supplementary Information [file srep13888-s1.doc]

**Comprehensive urinary metabolomic profiling and identification of potential noninvasive marker for idiopathic Parkinson’s disease**

Hemi Luan^1^, Liang-Feng Liu^2,3^, Zhi Tang^1^, Manwen Zhang^1^, Ka-Kit Chua^2,3^, Ju-Xian Song^2,3^, Vincent C.T. Mok^4^, Min Li^2, 3^*, Zongwei Cai^1^*

^1^ Department of Chemistry, Hong Kong Baptist University, Hong Kong SAR, China

^2^ School of Chinese Medicine, Hong Kong Baptist University, Hong Kong SAR, China

^3^ Mr. & Mrs. Ko Chi-Ming Centre for Parkinson's Disease Research, Hong Kong Baptist University, Hong Kong SAR, China

^4^ Department of Medicine and Therapeutics, Faculty of Medicine, The Chinese University of Hong Kong, Hong Kong SAR, China

* To whom correspondence may be addressed

**Name and complete address for correspondence:**

Prof. Zongwei CAI

Tel: +852-34117070; Fax: +852-34117348; E-mail: zwcai@hkbu.edu.hk

Address: 224, Waterloo Road, Kowloon Tong, Hong Kong SAR, China

Prof. Min LI

Tel: +852-34112919; Fax: +852-34112461; E-mail: limin@hkbu.edu.hk

Address: No.7 Baptist University Road, Kowloon Tong, Hong Kong SAR, China

Running Title: Metabolomic profiling of Parkinson’s disease


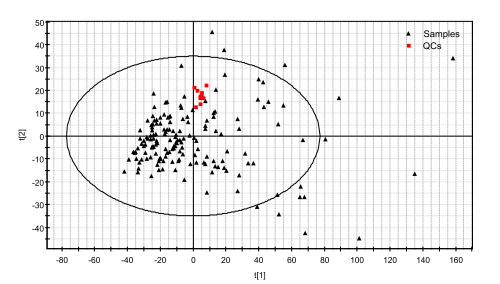


Figure S1 two dimensional principal component analysis (2D PCA) scores plot for consecutively analyzed QC samples in GC-MS platform. (Red indicates QC samples; Black, samples)


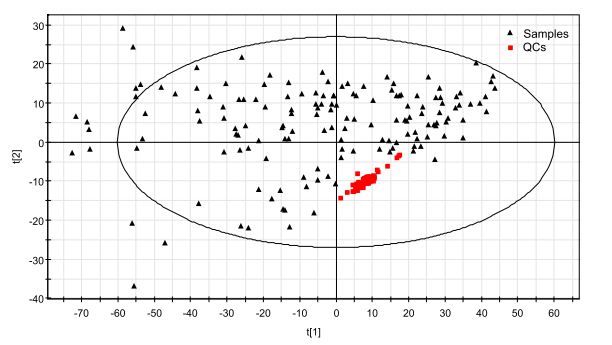


Figure S2 two dimensional principal component analysis (2D PCA) scores plot for consecutively analyzed QC samples in LC-MS platform. (Red indicates QC samples; Black, samples)

| Table S1 The common differential metabolites were identified in previous and present study |
| --- |
| **Metabolites** |
| Dihydrocortisol |
| 21-deoxycortisol |
| Cortisol |
| Hydroxyanthranilic acid |
| Xanthurenic acid |
| Hydroxytryptophan |
| Indolelactic acid |
| Kynurenine |
| Hexanoylglycine |
| Hydroxyphenylacetylglycine |
| Phenylacetylglycine |
| Furoylglycine |
| Tiglylglycine |
| Phenylacetylglutamine |
| Acetylphenylalanine |
| Hydroxyphenylacetic acid |
| Hypoxanthine |
| Urocanic acid |
| Imidazoleacetic acid |
| Trimethylamine N-oxide |
| Pyridoxic acid  Glycine |
